# Genomic Sequence and Experimental Tractability of a New Decapod Shrimp Model, *Neocaridina denticulata*

**DOI:** 10.3390/md12031419

**Published:** 2014-03-11

**Authors:** Nathan J. Kenny, Yung Wa Sin, Xin Shen, Qu Zhe, Wei Wang, Ting Fung Chan, Stephen S. Tobe, Sebastian M. Shimeld, Ka Hou Chu, Jerome H. L. Hui

**Affiliations:** 1 School of Life Sciences, The Chinese University of Hong Kong, Shatin, Hong Kong, China; E-Mails: nathanjameskenny@gmail.com (N.J.K.); yungwa.sin@cuhk.edu.hk (Y.W.S.); shenthin@163.com (X.S.); quzheouc@gmail.com (Q.Z.); wangweinbu@126.com (W.W.); tf.chan@cuhk.edu.hk (T.F.C.); kahouchu@cuhk.edu.hk (K.H.C.); 2 Department of Zoology, University of Oxford, Oxford OX1 3PS, UK; E-Mail: sebastian.shimeld@zoo.ox.ac.uk; 3 Department of Cell and Systems Biology, University of Toronto, Toronto M5S 3G5, Canada; E-Mail: stephen.tobe@utoronto.ca

**Keywords:** genomics, evolution, biotechnology, arthropods, crustaceans, decapod, shrimp

## Abstract

The speciose Crustacea is the largest subphylum of arthropods on the planet after the Insecta. To date, however, the only publically available sequenced crustacean genome is that of the water flea, *Daphnia pulex*, a member of the Branchiopoda. While *Daphnia* is a well-established ecotoxicological model, previous study showed that one-third of genes contained in its genome are lineage-specific and could not be identified in any other metazoan genomes. To better understand the genomic evolution of crustaceans and arthropods, we have sequenced the genome of a novel shrimp model, *Neocaridina denticulata*, and tested its experimental malleability*.* A library of 170-bp nominal fragment size was constructed from DNA of a starved single adult and sequenced using the Illumina HiSeq2000 platform. Core eukaryotic genes, the mitochondrial genome, developmental patterning genes (such as Hox) and microRNA processing pathway genes are all present in this animal, suggesting it has not undergone massive genomic loss. Comparison with the published genome of *Daphnia pulex* has allowed us to reveal 3750 genes that are indeed specific to the lineage containing malacostracans and branchiopods, rather than *Daphnia*-specific (*E*-value: 10^−6^). We also show the experimental tractability of *N. denticulata*, which, together with the genomic resources presented here, make it an ideal model for a wide range of further aquacultural, developmental, ecotoxicological, food safety, genetic, hormonal, physiological and reproductive research, allowing better understanding of the evolution of crustaceans and other arthropods.

## 1. Introduction

**Figure 1:**
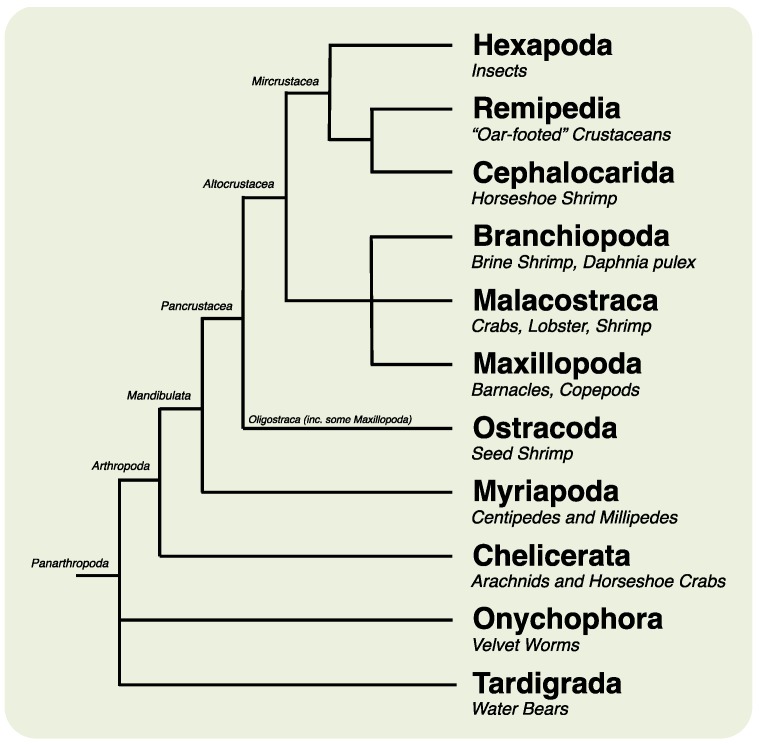
Simplified pancrustacean phylogeny, after [5]. Note some smaller, cryptic clades are not shown, including some members of the Maxillopoda, which are paraphyletically grouped with the Ostracoda (e.g., Branchiura and Pentastomida). Phylogenetic analysis is presently conflicted on the closest sister group to the Hexapoda: [5] places the Xenocarida as the outgroup to the Hexapoda, whereas [6] places the Branchiopods in this position.

Crustaceans are found worldwide in marine and terrestrial environments and are of great scientific and commercial importance. However, they are relatively underrepresented at the genomic level [1,2]. The Crustacea is conventionally divided into six classes [3], the Branchiopoda, Cephalocarida, Maxillopoda, Ostracoda, Remipedia and Malacostraca (which includes decapods, isopods, amphipods and stomatopods) (Figure 1), with an approximate number of 67,000 described living species [4]. Recent phylogenetic investigation has revealed that the Hexapoda, a group that includes the Insecta, is in fact nested within the crustaceans [5,6]. This renders the subphylum “Crustacea” paraphyletic, and the number of extant insect species is excluded from the number of crustacean species given above.

Extant published sequence resources in the Crustacea outside the Insecta are limited to that of a branchiopod, *Daphnia pulex* [7]. Although this animal is an important ecotoxicological model, its genome exhibits apparently high levels of gene duplication and loss and thus is not always suitable for use as an outgroup for comparison to the Insecta. Further, a single genome in the Crustacea also severely limits the conclusions that can be drawn as to the gain and loss of characters across the Pancrustacea, as current transcriptomic and expressed sequence tag (EST) resources will always only reflect the transcriptomic content of the specific tissues and time points sampled [1]. The provision of additional models to compare within the Crustacea is therefore a priority, given the importance of arthropods to the economy and a range of scientific spheres of investigation.

Malacostraca contains a number of orders, including the Amphipoda, Isopoda and Decapoda. A species within the Amphipoda, *Parhyale hawaiensis*, has already been used in developmental investigations [8], and isopods are familiar, due to the ubiquity of the common woodlouse, which is often used as a behavioural and environmental model [9]. The most economically important malacostracan crustaceans, however, belong to the order Decapoda. Decapods are both wild-caught and farmed and provide an important global food source. They are also ecologically vital as detritovores for environmental stability [10,11]. With an estimated 15,000 living crab, crayfish, shrimp, lobster and related species [12], the diversity of body plans and novelties seen in the Decapoda, including appendages, feeding mouthparts and segments, make them interesting models to study in evolutionary and developmental biology.

The cherry shrimp, *Neocaridina denticulata* (De Haan, 1844), is suggested as an excellent laboratory model within Decapoda—an experimentally tractable [13,14,15], cosmopolitan [16] and phylogenetically well-placed species [17]. It also has a limited history of use as a crustacean ecotoxicological model [13,14] and as a model of recent invasion [18]. The draft genome sequence of *N. denticulata denticulata* (De Haan, 1844) is presented here as a resource of benefit to a wide range of scientific investigations, including genomic, developmental, ecotoxicological, evolutionary, physiological and reproductive research. As another outgroup to the Insecta, it provides another lineage to that of the water flea, *D. pulex*, for comparison, and will allow further understanding of crustacean and arthropod biology. In addition, this species can be easily cultured and maintained in the laboratory [15], where insights gleaned from this species will be applicable to marine decapods and of much commercial utility for crustacean aquaculture and food safety. As such, this genomic resource will be of service to a range of scientific investigations worldwide.

## 2. Results and Discussion

### 2.1. Animal Culture and Lifecycle

**Figure 2:**
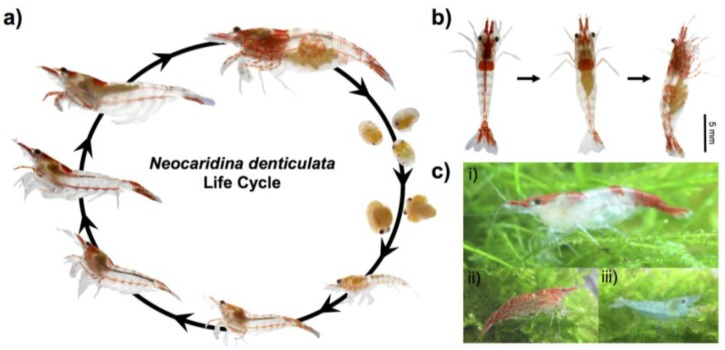
(**a**) The lifecycle of *N. denticulata*; and (**b**) the appearance of gravid female (**left**, dorsal view; **right**, side view) compared to a female without eggs (**centre**, dorsal view). The scale bar represents 5 mm on three adult shrimp in (**b**). (**c**) Some of the colour forms available commercially. (**i**) Red patched; (**ii**) punctate red patterning; and (**iii**) blue.

*Neocaridina* shrimp are native to many freshwater areas of East and Southeast Asia, tolerant of a wide range of conditions, commercially used for human consumption as a food flavouring agent and include a variety of colour forms (Figure 2c), and have spread around the world courtesy of the aquarium trade [19]. We have chosen *N. denticulata* for further study as it has much potential to become a model for aquacultural, developmental, ecotoxicological, food safety, genetic, hormonal, physiological and reproductive studies in the laboratory.

The start-up of culture and maintenance of *N. denticulata* is straightforward. Animals can be acquired commercially from the aquarium trade in a variety of colour morphs (Figure 2c). They can be kept in small freshwater tanks at room temperature, with simple filtration and aeration facilities sufficient for survival and reproduction. The native environments of *N. denticulata* generally are medium-soft and slightly alkaline (pH 7–7.5). However, *N. denticulata* can survive in a pH of 6.5–8.0 [14,19,20]. Optimum water temperature is 22–24 °C, and while shrimp are tolerant of a range of five degrees above and below this [15], temperatures should not be allowed to change rapidly or markedly. Low-powered filtration systems are recommended to aid the survival of juvenile shrimp, and a sponge filter is adequate, provided uneaten food is removed from tanks at regular intervals. Similar to other arthropods, *N. denticulata* is, however, sensitive to heavy metals and the use of insecticides, and care should be taken not to expose the culture tanks to airborne pollutants [15]. *N. denticulata* will thrive on a variety of foodstuffs [19], and we have had good results with several commercially available shrimp feed formulations. The provision of hiding spaces or plant material, such as Java Moss (Hypnaceae) is recommended, as decapods are known to be cannibalistic of tank mates during ecdysis [21], and plants can provide an alternative food source for the shrimp, both directly and as a substrate for algal growth.

Sexual maturity of the female can be observed through the transparent carapace and body instead of sacrificing the animal to measure the gonadosomatic index (ovary weight divided by total body weight) (Figure 2b). Breeding occurs in summer in subspecies in colder climates, whereas warm-weather subpopulations breed year-round [19]. Mating occurs shortly after ecdysis, following which the female lays her eggs, fertilizing them as they are laid; they are then attached to her pleopods (swimmerets). Approximately 20–30 eggs with sizes ranging from 0.57 to 1.08 mm [22] are laid simultaneously and are carried externally [15], where they are amenable to injection or manipulation (see Section 2.8). The eggs hatch at around 30 days post-mating, depending on water temperature [23]. *N. denticulata* grow to 7.3–28.5 mm in length, with both male and female *N. denticulata* attaining sexually maturity at 4–6 months of age [15,22]. Generation time is therefore relatively short compared to other shrimps or decapods and is similar to that of other model organisms, such as zebrafish and medaka, but faster than that of the frog, *Xenopus laevis*.

### 2.2. Genomic Sequencing

Genomic DNA from a single adult *N. denticulata denticulata* (De Haan, 1844) was extracted and sequenced on a single lane of the Illumina HiSeq2000 platform, as described in the experimental section. Basic read metrics relating to this sequencing are shown in Table 1. FastQC was run to ascertain read quality, with excellent results, and median Phred quality scores greater than 34 through to the last base in both reads (Supplementary Figure S1). No over-represented sequences were detected in our analysis. Raw sequence data have been uploaded to NCBI’s SRA (Bioproject PRJNA224755, Biosample SAMN02384679, experiment SRX375172, reads SRR1027643). After an initial assembly trial, Bowtie [24] was used to determine actual fragment size and the standard deviation for future use.

**Table 1:** Basic metrics relating to raw reads.

Platform	Illumina HiSeq2000
Number of Reads	364,013,140
Read Length (bp)	100
Average GC %	36
Fragment Size	167.22
Fragment Size SD (bp)	12.01

### 2.3. Genomic Assembly

**Figure 3:**
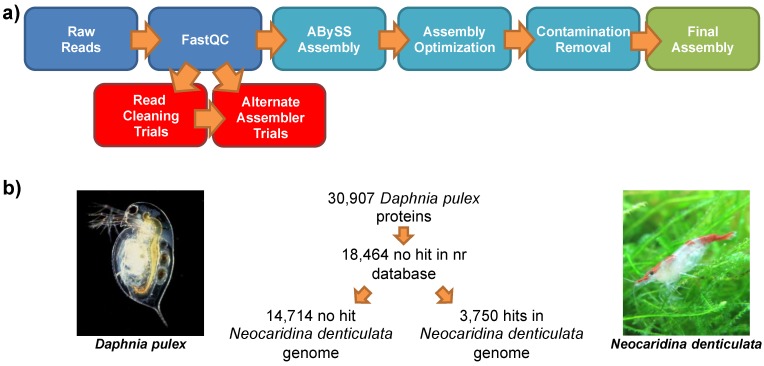
(**a**) Schematic diagram of the genomic assembly of shrimp *N. denticulata.* (**b**) The summary statistics relating to the comparison of *D. pulex* and *N. denticulata* genomes, compared to the non-redundant (nr) database. *D. pulex* image is courtesy of Paul Hebert [32].

Genomic assembly procedures are summarized in Figure 3a. After initial trials using a range of assembly software, including Velvet [25] and SOAPdenovo [26], raw reads were assembled using the abyss-pe script in ABySS [27] with a *k-*mer size of 51. Read cleaning using Sickle [28] and Musket [29] was assayed, but found to impair assembly by conventional metrics. Results of the empirically-determined “best” assembly are shown in Table 2. This assembly can be downloaded from [30] or can be supplied by the authors upon request.

While the genome size of *N. denticulata* has not been measured, a closely related species in the same family, Atyidae, *Antecaridina* sp., has been determined to have a *C*-value of 3.30 pg, or approximately 3.2 Gbp [31]. Such large genomes are known to be difficult to assemble and traditionally exhibit a large amount of repetitive sequences. Our efforts have recovered sequences totalling 1.2 Gbp. If the genome size of *N. denticulata* is close to 3 Gb, one possibility could be that the short fragment length used for library construction constrains the contiguity of our sequences across repetitive regions and, thus, also accounts for the relatively short N50 (Table 2). Assuming a 3 Gb genome, our sequence data provide approximately 12x coverage. A small amount of contamination with bacterial DNA (three large contigs greater than 30 kb in length, similar to the *Novosphingobium* sp. bacterial DNA sequence) without high similarity to the known *Wolbachia* sequences was removed manually after BLASTN detection. The availability of funding for additional long mate pair data for scaffolding in the future would greatly enhance contiguity and allow the exploration of the content of non-coding regions, which we suspect are poorly recovered in this assembly.

**Table 2:** Metrics relating to final assembly.

Criteria	Value (base pairs)
Min. contig length	200
Max. contig length	124,746
Mean contig length	383.84
Standard deviation of contig length	285.33
Median contig length	302
N50 contig length	400
Number of contigs	3,346,358
Number of contigs ≥1 kb	97,432
Number of contigs in N50	987,201
Number of bases in all contigs	1,284,468,468
Number of bases in contigs ≥1 kb	132,397,543
GC Content of contigs (%)	35.21

### 2.4. Comparison of Core Eukaryotic Genes

Despite the scaffold size being relatively short, our data contain a great deal of useful information concerning the coding regions of this genome. We used the Core Eukaryotic Gene Mapping Approach (CEGMA) dataset [33], which consists of 458 single-copy genes found in almost every eukaryote genome, as an assay of the completeness of the coding sequence coverage contained in our sequence data. Using TBLASTN [34] with a cut-off of 10^−3^, of the 458 genes, only three (ribosome biogenesis protein RLP24, ribosomal 60S subunit protein L24A and the HSP binding protein, YER156C) did not possess a recognizable hit in our sequence. This *E*-value cut-off was selected empirically after several trials and, at this stringency, represents 455 genes or 99.3% recovery of the expected coding sequences, which suggests that our dataset is excellent as a starting point for assaying the presence of genes in decapod crustaceans. Of these contigs annotated with CEGMA, the mean size of the contigs identified is 2500.01 bp and the median is 534 bp, which are longer than our N50 and mean/median contig sizes.

### 2.5. Mitochondrial Genomic Characteristics

**Figure 4:**
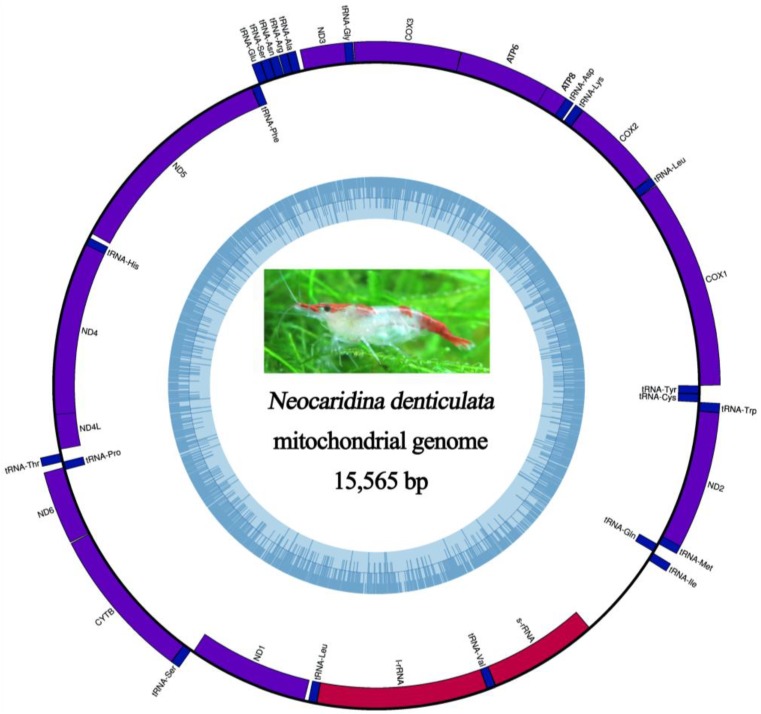
The *N. denticulata* *denticulata* mitochondrial genome. The orientation of genes is represented by the position on outside circle (transcription clockwise or anticlockwise is represented outside or inside the form, respectively). Local nucleotide identity (GC, dark blue) is represented on the inner ring as implemented by OrganellarGenomeDRAW (OGDRAW) [36].

Retrieval of the *N. denticulata* *denticulata* (Crustacea: Caridea) mitochondrial genome from our dataset in a single, well-assembled contig revealed a circular molecule of 15,565 bp that encodes the typical set of 37 metazoan genes (13 protein-coding genes, 22 transfer RNA genes and two ribosomal RNA genes). The majority-strand (α) and minority-strand (β) encode 23 and 14 genes, respectively (Figure 4 and Supplementary Information: Table S1 in mtDNA). The result is comparable to the mitogenome of a related subspecies, *N. denticulata sinensis*, which differs by 4 bp in length and has slight differences in amino acid and codon usage [35]. Due to the compactness of the mitochondrial genome, ten instances of gene overlaps were found. A total of 841 bp non-coding nucleotides were found, with 153 bp in 13 intergenic regions and a 688 bp-long non-coding region between the *srRNA* and *trnIle* (Supplementary Information: Table S1 in mtDNA).

The typical metazoan initiation codon for transcription “ATN” is used by 12 out of 13 protein-coding genes, whereas *cox1* employs “AAG” as the start codon, which is similar to other caridean mitochondrial genomes (Supplementary Information: Table S2 in mtDNA). The open-reading frames of 11 protein-coding genes are terminated by the typical stop codon (TAA or TAG), while the remaining two genes (*cox2* and *nad4*) have an incomplete stop codon “T-”. All protein-coding genes and both rRNAs have skewed T *vs.* A (AT skew ranging from −0.041 to −0.293). The majority of protein-coding genes have a skew of C *vs.* G, but both rRNAs have a skew of G *vs.* C (the GC skews are 0.316 and 0.273 for *srRNA* and *lrRNA*, respectively) (Supplementary Information: Table S3 in mtDNA). In total, there are 3696 codons in all 13 mitochondrial protein-coding genes, excluding incomplete termination codons, and the most frequently used amino acids are Leu (15.58%), followed by Ser (9.60%), Ile (8.39%), Phe (8.12%) and Val (7.06%) (Supplementary Information: Table S4 in mtDNA).

**Figure 5:**
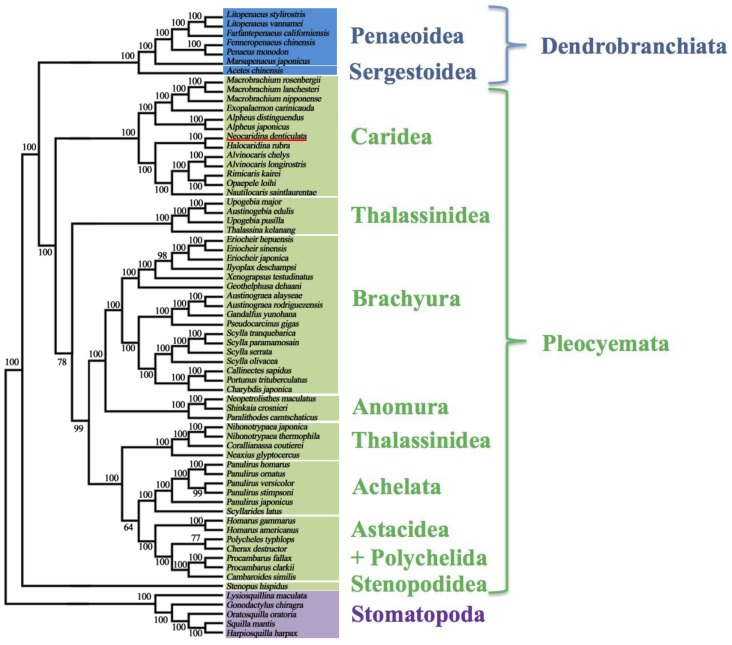
The Bayesian phylogenetic tree showing inter-relationships of a variety of malacostracan crustacean species, including the position of *N. denticulata* (underlined in red), based on concatenated nucleotide sequences from mitochondrial genomes. Numbers at nodes represent the posterior probability expressed out of 100.

Using the nucleotide sequences of the mitogenome of *N. denticulata denticulata* presented here, a Bayesian phylogenetic reconstruction of malacostracan inter-relationships was performed and summarized in Figure 5, which reinforces our knowledge of the inter-relationships of the Caridea.

**Figure 6:**
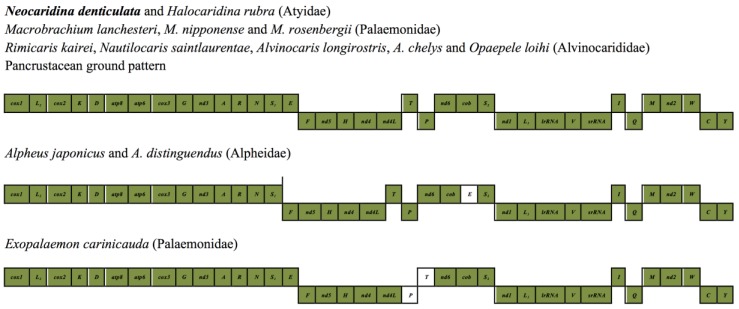
*N. denticulata* mitochondrial genome organisation compared to that of other crustaceans. *N. denticulata* possesses the stereotypical pancrustacean mitochondrial gene order as described first in *Limulus polyphemus* [38]; the orders of closely related species are provided for ease of comparison.

Further in our analysis, the arrangement of the *N. denticulata* mitochondrial genes is found to be identical to the hypothetical pancrustacean ground pattern (Figure 6), whereas some other members of infraorder Caridea, such as *Exopalaemon carinicauda* (Palaemonidae), *Alpheus japonicus* and *A. distinguendus* (Alpheidae), all have their mitochondrial gene orders rearranged ([37]; Figure 6).

### 2.6. Recovery of Hox Genes and Other Families

**Figure 7:**
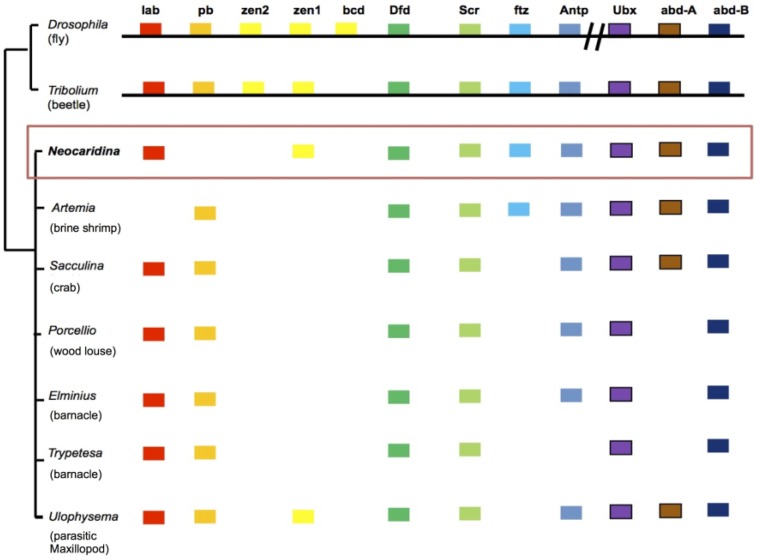
Hox cluster gene recovery in *N. denticulata*, compared to a number of other pancrustacean species (diagram after [39]). Sequences provided in Supplementary Information, HoxL tab.

To confirm the coverage of the coding regions of this genome, several well-annotated and catalogued developmental gene families were assayed. Our searches suggest that most, if not all, of the coding regions of the genome were recovered in our assembly. For example, Hox genes pattern the developing anteroposterior axis of animals, and 12 families of Hox genes are commonly described in pancrustaceans [39]. In our analysis, nine of the 12 Hox gene members could be identified (Figure 7). Of the three missing families, *zen2* and *bcd* have not been identified in any crustaceans outside the Insecta to date [39], so their absence in our dataset very probably indicates that these genes are insect novelties. Only one Hox gene absent from our dataset, *pb*, could be a consequence of the poor recovery of this genomic locus or the first loss of this gene reported in the Crustacea *sensu stricto*. The identification of Hox gene *zen1* in *N. denticulata* provides the first identification of this gene in the decapods (Figure 7). Unfortunately, these sequences are predominantly found on short contigs (see Supplementary Information: HoxL tab), and therefore, no syntenic relationship information can be gleaned from the data as it stands.

Similarly, our recovery of other families of well-catalogued genes is equally impressive (Table 3). The Fox genes, which are helix-turn-helix genes with an 80 to 100 amino acid “Forkhead Box” motif, are separated into 23 classes, which perform a variety of roles in metabolism and embryonic development [40,41]. We find 21 homologues of these genes in our dataset, from 16 families, with almost all those missing probably restricted to clades to which *N. denticulata* does not belong and, hence, was not expected to be found in the genome sequence data. The one exception to this is *Fox L1*, which appears to be absent from our dataset, while being described in other protostomes.

T-box genes are also well-catalogued and perform a similarly wide range of vital evolutionarily conserved roles [42]. Of these genes, we recovered 10 sequences corresponding to five families. The families missing, *T-Brain*, *Tbx4/5* and *Tbx 15/18/22*, are genes limited to other Superphyla [42,43,44] within the Metazoa and, hence, are not expected to be found in *N. denticulata*.

Another gene repertoire examined to show the conservation of key pathways was the core microRNA/miRNA processing cassette [45]. Sequences (such as *Ago*, *Dicer*, *Exportin-5*) from the totality of the expected pathway were recovered. These findings further suggest that the coding sequences of the *N. denticulata* genome are well-recovered in our genomic build.

**Table 3:** Recovery of developmentally important gene families. Details and sequences provided in Supplementary Information. Missing genes are generally absent ancestrally, rather than in our assembly, as discussed in the main text.

Gene Classes	Homologues Recovered	Missing
Homeobox Genes (HoxL)	20	Pb
Fox Genes	21	Fox E, H, I, J2/3, L1, M, Q1
T-box Genes	10	T-Brain, Tbx 4/5, Tbx 15/18/22
miRNA processing genes	8	-

### 2.7. Comparison to the Lineage-Specifically Gained Genes of Daphnia Pulex

To gain an understanding of crustacean genome evolution, we compared the genome composition of *N. denticulata* with that of the branchiopod, *D. pulex*, the only publically available crustacean genome sequence to date. The genome of *D. pulex* is noted for its large number of gene duplications and a rapid rate of genomic evolution, and a previous study has suggested 17,424 new and 1079 lost genes in the branch leading to *D. pulex* [7].

To determine whether the genes gained in *D. pulex* represent a Crustacea-wide gain or are truly limited to *D. pulex* alone, we used BLASTX to compare the *D. pulex* proteome to the *N. denticulata* genome (Figure 3b). As significant numbers of sequences have been added to the non redundant (nr) database since the publication of the *D. pulex* genome in 2011, the 30,907-sequence proteome of *D. pulex* was blasted against the nr database using Blast2GO (database as of October, 2013, BLASTP, cut-off e^−6^, crustacean genes (excluding the Insecta) excluded from blast hits). At this threshold, 18,464 *D. pulex* genes were found to have no hits in nr.

The *D. pulex* proteome was then compared to our genomic build using BLASTX. As *E*-values cannot be directly compared between datasets, as they depend on the size of the database and the search used, and a number of *E*-values were trialled for the initial comparison of the *D. pulex* proteome and *N. denticulata* genome. By way of example, 16,640 *D. pulex* genes were found to have no hits in *N. denticulata* at an *E*-value of 10^−3^, whereas 18,739 *D. pulex* genes have no hits at an *E*-value of 10^−6^. As this latter figure represented a similar number to the unannotatable gene complement of *D. pulex*, this was taken as our cut-off for further comparison.

Of the 18,464 *D. pulex* genes with no hit in the nr database, 14,714 had no identifiable homologue in shrimp either. However, 3750 *D. pulex* genes, unidentifiable previously, were found to have hits in *N. denticulata* above the cut-off threshold of an *E*-value of 10^−6^ (Figure 3b). These 3750 genes may represent novelties gained in the maxillopod, branchiopod and malacostracan lineages or pancrustacean genes lost in sequenced insects. These genes will be key targets for future work on crustacean novelties and are likely of interest to a range of researchers. The list of 3750 *D. pulex* genes, along with the lists of all other genes and appropriate sequences, are summarized in Supplementary Information, under the Details of Hits and Sequences tabs.

Additionally, 1927 of the 12,443 *D. pulex* genes identifiable in the nr database have no hit in the shrimp, which either represents genes not assembled in our dataset or losses on the malacostracan lineage leading to *N. denticulata*.

### 2.8. MS-222 Treatment

In addition to describing the culture and sequencing of the draft genome of *N. denticulata*, we have also established an anaesthesia technique using tricaine methanesulfonate (MS-222) for future genetic manipulation requiring microinjection of eggs or adults.

**Figure 8:**
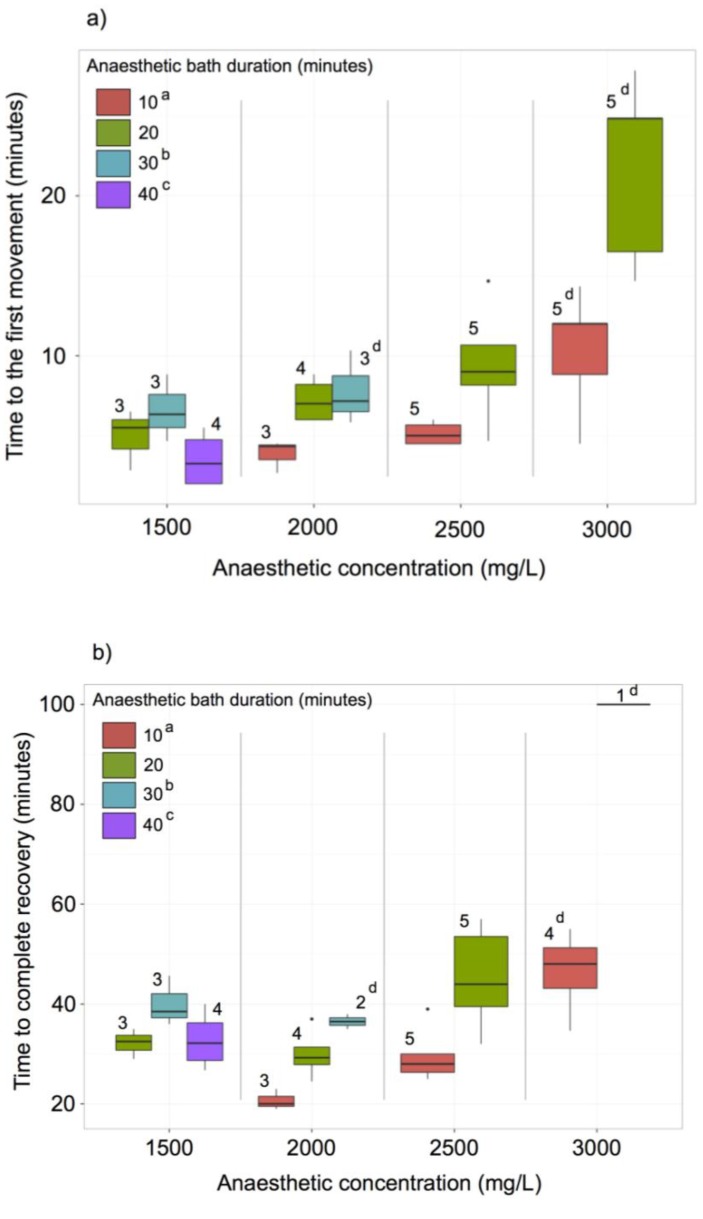
Boxplot of recovery time when (**a**) the first movement and (**b**) complete recovery was observed in *N. denticulata* individuals after anaesthesia in different MS-222 concentrations and bath durations. The horizontal line shows the median, boxes the first and third quartiles, whiskers the highest and lowest values that are within the 1.5 inter-quartile range, black dots the outliers and numbers above boxes the sample size. ^a^ A ten-minute bath duration is insufficient to induce anaesthesia at a concentration of 1500 mg L^−1^. ^b^ A thirty-minute bath duration is lethal to some individuals at a concentration of 2000 mg L^−1^ and was not performed for higher concentrations. ^c^ The forty minute bath duration was not performed for concentrations higher than 1500 mg L^−1^. ^d^ Some individuals that showed signs of movement were not fully recovered at the end of the assayed period.

The average induction time for the adult shrimp in 1500, 2000, 2500 and 3000 mg L^−1^ baths of MS-222 were 27 min 5 s (Standard error (SE) = 3 min 41 s), 12 min 42 s (SE = 1 min 15 s), 10 min 58 s (SE = 53 s) and 6 min 27 s (SE = 47 s), respectively (Figure 8). As only two out of ten individuals entered anaesthesia in 1000 mg L^−1^ bath of MS-222 after 40 min, we concluded this concentration is too low to induce anaesthesia in *N. denticulata*, and no further analysis was performed at this or a lower concentration. Both the anaesthetic concentration and bath duration clearly affect the recovery time of *N. denticulata* (Figure 8a,b). At higher MS-222 concentrations, shrimp took a longer time for their first movement (Figure 8a; General Linear Model (GLM): F_3,30_ = 26.1, *p* < 0.001) and complete recovery (Figure 8b; GLM: F_3,24_ = 21.4, *p* < 0.001) was observed. Similarly, a longer bath duration also led to a longer time to their first movement (Figure 8a; GLM: F_3,30_ = 11.0, *p* < 0.001) and complete recovery (Figure 8b; GLM: F_3,24_ = 16.8, *p* < 0.001). The effect of bath duration was more obvious in higher rather than in lower MS-222 concentrations (the interactive effect of concentration and duration on time until first movement: F_3,30_ = 3.2, *p* < 0.05). At a concentration of 3000 mg L^−1^ MS-222, one/four individuals of five sampled were dead after 10 and 20 min of anaesthetic bath, respectively. Therefore, a 3000 mg L^−1^ concentration is too high for safe anaesthesia in *N. denticulata*. Following the treatment of animals with 2000 mg L^−1^ for 30 min, one individual of the three sampled died, suggesting that a long bath duration could be lethal to more susceptible individuals, even at a lower anaesthetic concentration. In our experiments, all other individuals treated with MS-222 at less than 3000 mg L^−1^ could recover completely, and none died in the following three days.

To allow sufficient time for experimental manipulation, we have determined that bathing *N. denticulata* for 20 min in 2500 mg L^−1^ MS-222 is optimal for longest non-lethal dose and duration. Under these conditions, the recovery time until first movement is 9 min 26 s (SE = 1 min 38 s). Compared to other aquatic animals, the MS-222 concentration we suggest is higher than those for fish anaesthesia in general, which vary from 50–400 mg L^−1^ [46] However, the effective MS-222 concentration for crustacean anaesthesia is generally found to be high, for example, 500 mg/L on the microcrustacean *Eucypris virens* [47], and could vary among different crustaceans with 100 mg L^−1^ and 1000 mg L^−1^ MS-222 being found ineffective on the shrimp, *Macrobrachium rosenbergii* [48], Chinese mitten crab, *Eriocheir sinensis* [49], and crayfish, *Orconectes virilis* [50].

## 3. Experimental Section

### 3.1. Animal Husbandry and Genomic DNA Extraction

*N. denticulata denticulata* (red patched strain) were sourced from local suppliers and kept in a recirculating freshwater aquarium at room temperature, at approximately 25 °C. A single adult was starved for 2 days prior to gut dissection, and whole animal genomic DNA was extracted using DNeasy Blood & Tissue Kit (Qiagen, Hilden, Germany), according to the manufacturer’s protocol.

### 3.2. Illumina Hi-Seq and Assembly

The *N.* *denticulata* genomic DNA sample was sequenced on a single lane on the Illumina HiSeq2000 platform. DNA was prepared for sequencing using a TruSeq DNA Sample Preparation Kit by BGI Hong Kong. Reads were first filtered by BGI according to their internal protocol, including the removal of the adaptor sequence, contamination and low-quality reads. Raw sequence data were uploaded to NCBI’s SRA (Bioproject PRJNA224755, Biosample SAMN02384679, experiment SRX375172, reads SRR1027643). ABySS 1.3.3 [27] was used to assemble the genome at a *k*-mer length of 51 and all default settings. A minimum scaffold length of 200 bp was imposed after the assembly was complete.

### 3.3. mtDNA/ Nuclear Gene Retrieval

Gene sequences were identified using TBLASTN [34] searches using known gene sequences of confirmed homology downloaded from the NCBI nr database as queries. Genes thus putatively identified were then reciprocally blasted against the NCBI nr database using BLASTX to further confirm their identity. Characteristic domains and trees constructed in MrBayes 3.1 [51] using genes of known homology downloaded from the nr database and aligned using MAFFT [52] were used to further confirm the homology for developmental gene families (data not shown).

### 3.4. Gene Comparison

For the comparison of *D. pulex* and *N. denticulata* sequences, the *D. pulex* proteome (FilteredModelsv1.1.aa) was downloaded from the Joint Genome Institute (JGI) website [53]. Standalone ncbi-blast-2.2.23+ [34] and Blast2GO [54] were used to perform blasts locally, as described in the text, with the latter used only for the comparison of the *D. pulex* dataset with the nr database.

### 3.5. mtDNA Annotation and Display

The locations of the 13 protein-coding genes (PCGs) and 2 rRNAs were determined with Dual Organellar GenoMe Annotator (DOGMA) [55] and subsequent alignments with caridean mitochondrial genes. All tRNA genes were identified by tRNAscan-SE 1.21 [56]. The gene map of the mitochondrial genome was drawn by OrganellarGenomeDRAW (OGDRAW) [36]. Codon usage in 13 PCGs of the mitochondrial genome was estimated with DnaSP 5.10.1 [57]. The nucleic acid sequences of 13 PCGs were aligned using Clustal W [58]. To determine the best fitting model of sequence evolution for the nucleic acid dataset, a nested likelihood ratio test was performed using jModelTest 2 [59]. After the evolutionary model (GTR + G + I) was determined, the phylogenetic relationship was inferred by MrBayes 3.1 [51]. The Markov Chain Monte Carlo analyses were run for 1,000,000 generations (sampling every 1000 generations) to allow adequate time for convergence. After omitting the first 250 sampled trees as “burn-in”, the remaining 750 sampled trees were used to estimate the Bayesian posterior probabilities.

### 3.6. MS222 Anaesthesia

Anaesthesia was performed by dissolving MS-222 salt (Sigma, St. Louis, MO, USA) in culture tank water. Sodium bicarbonate was added to neutralize the pH to the original culture tank water pH (*i.e*., 7.2). Ten individual adult shrimp were tested for each of the five MS-222 concentrations (1000, 1500, 2000, 2500 and 3000 mg/L). Shrimp were kept in an aerated anaesthetic bath for durations as stated (10, 20, 30 or 40 min). The induction time (the time from immersion in the anaesthetic bath until the shrimp showed no movement and no reaction to touch stimuli) was recorded. After the anaesthetic bath, the shrimps were immediately put in aerated water from the original tank for recovery. The recovery time for each individual was also recorded, including: (a) the time until the first movement of pleopods and/or pereiopods; and (b) the time until complete recovery at which the individual resumed balanced movements and normal feeding behaviour. A general linear model (GLM) was used to test the recovery time difference between different anaesthetic conditions, with the recovery time being used as the dependent variable and anaesthetic concentration and duration as independent factors.

## 4. Conclusions

To date, genomic sampling in Crustacea remains depauperate, but resources such as the one presented here will aid in the further study of this neglected taxon. As suggested by the recovery of the majority of core eukaryotic genes, the complete and un-rearranged mitogenome and considerable fractions of several developmental gene cassettes, the *N. denticulata* sequences presented here represent an excellent crustacean model for comparison to other arthropods. Our analysis also allows the identification of 3750 putatively crustacean-specific genes that could shed light on the understanding of the crustacean genome evolution. A non-lethal anaesthesia protocol has also been established for use in future genetic manipulation of this species. Given the lack of an easily-cultivable decapod laboratory model, we propose the shrimp, *N. denticulata*, as an experimentally tractable, easily grown species for a wide variety of future investigations in aquacultural, developmental, ecotoxicological, evolution, food safety, genetic, hormonal, physiological and reproductive research of decapod crustaceans.

## Supplementary Files

Supplementary File 1 Supplementary Information (XLSX, 4729 KB)
